# Floating appendix: post-traumatic amputation of the appendix as sequela or complication?: a case report

**DOI:** 10.1186/s13256-021-02747-z

**Published:** 2021-04-07

**Authors:** Kanika Sharma, Shreya Tomar, Shilpa Sharma, Minu Bajpai

**Affiliations:** Department of Pediatric Surgery, All India Institute of Medical Sciences, New Delhi, 110029 India

**Keywords:** Appendiceal transection, Abdominal blunt trauma, Burst abdomen, Case report, Pediatric

## Abstract

**Background:**

Appendicitis following trauma is a well-documented sequela of blunt trauma to the abdomen, while appendiceal transection following trauma is extremely rare. Literature reports have documented appendicitis and appendiceal transection as the presenting pathology in a trauma setting. This is first report of auto-amputation of the appendix as a delayed presentation with peritonitis, which was detected during the second surgery in a child with blunt abdominal trauma.

**Case presentation:**

A 11-year-old Asian boy presented to our center with a 2-day history of blunt abdominal trauma and chief complaint of severe abdominal pain. On evaluation, a computed tomography scan showed gross pneumoperitoneum. The child underwent emergency laparotomy, where a jejunal perforation was noted, which was repaired. The rest of the bowel and solid organs were healthy. The child was managed in the intensive care unit postoperatively, when he developed a burst abdomen. During the second surgery, pyoperitoneum and free-floating appendix were found in the left paracolic gutter. After peritoneal wash, the bowel was noted to be healthy and the previous jejunal repair was intact. The child was allowed oral intake of food and discharged on postoperative days 4 and 8, respectively. At the 1-year follow-up, he remained asymptomatic.

**Conclusions:**

This case report is unique as it describes auto-amputation of the appendix as a delayed event in the course of treatment for blunt trauma of the abdomen. Although a remote event, the possibility of amputation of the appendix should be retained as a differential diagnosis and unusual complication in cases of delayed peritonitis.

## Background

Most pediatric abdominal injuries are from blunt trauma following road traffic accidents or falls. Solid-organ injuries are far more common than hollow viscous injury [1]. The incidence of appendicitis following blunt abdominal trauma is reported in the literature; however, reports of appendiceal transection are uncommon [2, 3]. Based on a literature search, there are no documented cases of auto-amputation of the appendix as a delayed event, which is reported in the present case.

## Case presentation

A 11-year-old Asian boy presented to a tertiary-level trauma center with complaints of severe abdominal pain for 2 days after a fall from a bicycle. There was no history of nausea, vomiting, abdominal distension or fever. On examination, the child was conscious and oriented, with a normal breathing pattern. Assessment of vital signs revealed that he was afebrile, heart rate was 120 beats per minute (tachycardia for age), respiratory rate was 20 breaths per minute, and blood pressure in the supine position was 110/70 mmHg. Rhythmic pulse with good volume was palpable at all examination sites. No signs of external injuries were noted. All limbs were able to move freely in all directions with complete range of motion. Abdominal assessment revealed a slightly elevated temperature, with contours maintained and no abdominal distension; guarding and rigidity were present, and deep palpation was not possible.

**Fig. 1:**
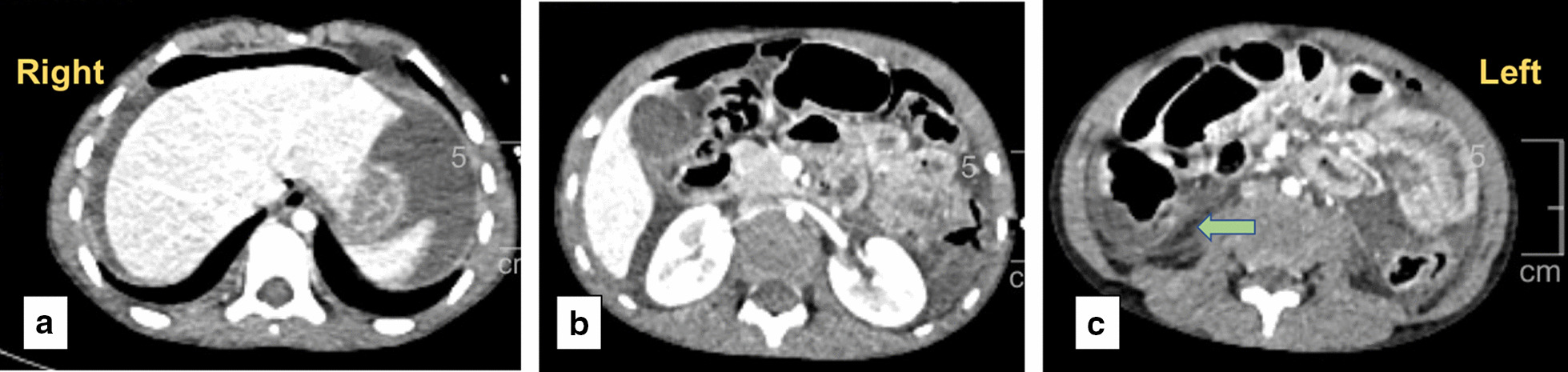
Contrast-enhanced computed tomograph (CECT) of the torso done in the emergency department showing the gross pneumoperitoneum (**a**) and inter-loop free air (**b**). The intact appendix in the right iliac fossa is identified and marked with an arrow (**c**).

Focused assessment with sonography in trauma (FAST) showed the presence of free fluid in the hepatorenal pouch. Subsequent contrast-enhanced computed tomography (CECT) of the torso demonstrated pneumoperitoneum with thickening of the proximal jejunal loops (Fig. 1a, b). Blood test results showed hemoglobin of 8.9 g/dL and total leucocyte count of 12,700/mL In view of the pneumoperitoneum, exploratory laparotomy was performed. Intraoperatively, 200 mL of purulent peritoneal fluid was noted, with a 2 cm jejunal perforation located 30 cm from the duodenojejunal (DJ) junction. Primary repair of the perforation was performed with generous peritoneal lavage, and a drain was placed in Morrison’s pouch. The rest of the bowel, along with the ileocecal region, was examined and was found to be healthy.

**Fig. 2:**
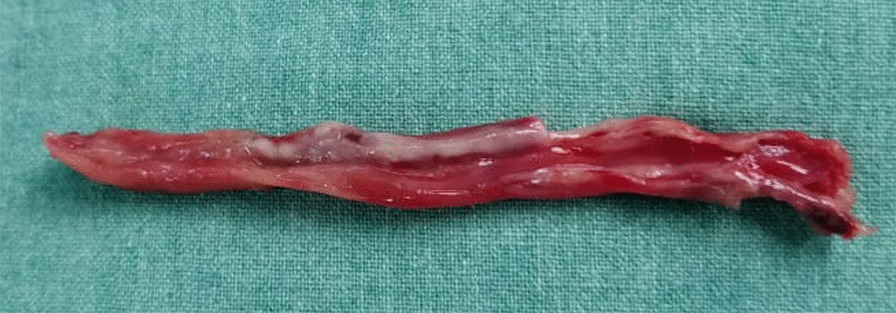
Tubular appendix specimen (dissected longitudinally) retrieved from left paracolic gutter during the second surgery

The child was monitored in intensive care, where vital signs were stable and electrolytes were within normal limits. He was continued on no oral intake of food or fluids, as he had passed flatus only. However, on postoperative day (POD) 6, the child developed fever with increasing abdominal girth. Serosanguinous discharge was noted from the drain site. On POD 7, serosanguinous discharge was noted at the wound site as well, followed by bluish discoloration and thickening of the skin around the wound. The peripheries were warm and pink with palpable lower limb pulses. A few hours later, wound dehiscence was noted. The child was then taken to the operating room, where exploratory laparotomy was performed. Intra-operatively, purulent peritoneal fluid was observed with flimsy inter-bowel loop adhesions, for which gentle adhesiolysis was done. A tube-like structure, suspected to be free-floating appendix, was noted in the left paracolic gutter (Fig. 2). The previous jejunal repair was intact. Examination of the rest of the bowel showed that it was healthy. However, erythema was present around the ileocecal junction, and no appendiceal stump was noted around it. Pus flakes were noted in all quadrants, for which generous peritoneal lavage was performed. The drain in Morrison’s pouch was replaced.

The child was stable during the postoperative period. On POD 4, he passed flatus and stool, following which he was allowed oral intake of food which was built up gradually to full diet over 5 days. The nasogastric tube and abdominal drain were removed on POD 3 and POD 5, respectively. The child was discharged on POD 8 on full diet following the second surgery.

The histopathology report of the tubular structure confirmed it to be appendix with areas of necrosis, acute inflammatory infiltrates and focal areas of hemorrhage. On retrospective evaluation of the initial CECT, the appendix was confirmed and localized in the right iliac fossa at the ileocecal junction (Fig. 1c).

The child has been followed up for a year now and has been asymptomatic.

## Discussion

The normal appendix is a small tube-like structure which is attached to the mesentery at one point, thus having a wide range of mobility around its axis. Also, the periappendiceal fat and adjoining paracolic gutter help the appendix escape most of the trauma from abdominal injuries. However, the narrow lumen and an end arterial blood supply make it susceptible to trauma. While appendicitis can independently lead to amputation of the appendix, trauma is a rare but reported cause of appendicitis [4]. A review of the literature showed that pediatric cases of traumatic appendiceal amputation were in response to the primary impact of the injury, which may or may not occur as sequelae with traumatic appendicitis [5, 6]. However, the present scenario was different in terms of the occurrence of appendicular amputation as a post-trauma, postoperative event, further leading to its complications.

Small bowel perforations near the ligament of Treitz in children are most often attributed to mesenteric or small bowel avulsion. These injuries are often diagnosed late because of nonspecific symptoms [1]. This was the case in our patient, who presented to the hospital 2 days after injury. However, the presence of a jejunal tear at presentation, which was efficiently repaired, leading to subsequent auto-amputation of a previously normal appendix in the postoperative period is unusual.

Appendiceal transection is seen in cases of seat-belt injury, where the compression and deceleration forces cause an increase in intraluminal pressure [7]. In children, handle-bar injuries are also a leading cause of appendiceal transection [5, 8]. Although the mechanism of injury in the present case was reported to be a simple fall from a bicycle, handle-bar injury could not be ruled out. Appendicitis may be missed or diagnosed late in a setting of co-existent trauma. Thus, it may often be presented as complicated appendicitis with peritonitis and appendiceal perforation. The pathophysiology of appendicitis in trauma is well described by Etensel *et al*., where a vicious cycle of increased intra-abdominal pressure and visceral edema leads to appendicitis [3]. The most probable theory for appendiceal transection in the present case is the associated abdominal compartment syndrome, where ischemic injury and compression injury to the appendicular base may have occurred, leading to auto-sloughing and auto-amputation. The burst abdomen can also be attributed to abdominal hypertension. The other proposed hypotheses are as follows:

1. Hyperviscosity of the mesenteric blood flow across the end appendicular artery with possible hypovolemia, which could have been ongoing during the delayed presentation.
2. Mesenteric spasm due to injury, where the blood to the rest of the bowel was restored due to the dense network of blood vessels in the mesentery; however, the end artery, like the appendiceal artery, had a defense mechanism recruited which led to further inflammation and edema at the base, leading to auto-amputation of the appendix.
3. Due to injury and surgery, the body was already under tremendous stress, with numerous inflammatory markers circulating in the bloodstream, which could excite an inflammatory cascade in the appendix.
4. Inadvertent trauma to the ileocecal junction during the first surgery.

The presence of peritonitis and burst abdomen suggests that postoperative appendicitis led to complicated perforated suppurative peritonitis, which can be attributed to abdominal hypertension or vice versa. However, the early intervention and close monitoring helped to preclude further morbidity or complications. Therefore, a high level of suspicion and appropriate observation and examination should be the key management strategy in the postoperative period for all patients with blunt abdominal trauma.

Unlike the reported cases of appendiceal transection, which represent the primary pathology following blunt trauma of the abdomen, the present case can be considered as a delayed or secondary pathology for peritonitis in an acute trauma setting. The case addresses the unique situation of auto-amputation of the appendix as a delayed event in the course of treatment of blunt abdominal trauma, its unusual complication of post-traumatic postoperative abdominal compartment syndrome and delayed pathology for peritonitis in an acute trauma setting. Although a remote event, the possibility of amputation of the appendix should be retained as a differential diagnosis and unusual complication in cases of delayed peritonitis. Thus, this case highlights the need for trauma care surgeons to be more vigilant during surgery and during postoperative care.

## Conclusion

Traumatic amputation of the appendix is a rare possibility in blunt abdominal trauma; however, when missed, it can have serious complications. Secondary traumatic amputation of the appendix following the primary injury can be an independent identity. Therefore, a thorough inspection of the peritoneal cavity and soft tissue is essential during exploratory laparotomy.

## Data Availability

Not applicable.
